# Industrial Waste Utilization of Carbon Dust in Sustainable Cementitious Composites Production

**DOI:** 10.3390/ma13153295

**Published:** 2020-07-24

**Authors:** Mohammad R. Irshidat, Nasser Al-Nuaimi

**Affiliations:** Center for Advanced Materials (CAM), Qatar University, P.O. Box 2713, Doha, Qatar; anasser@qu.edu.qa

**Keywords:** carbon dust, industrial waste, cement mortar, strength, microstructure

## Abstract

This paper experimentally investigates the effect of utilization of carbon dust generated as an industrial waste from aluminum factories in cementitious composites production. Carbon dust is collected, characterized, and then used to partially replace cement particles in cement mortar production. The effect of adding different dosages of carbon dust in the range of 5% to 40% by weight of cement on compressive strength, microstructure, and chemical composition of cement mortar is investigated. Scanning electron microscopy (SEM), X-ray diffraction (XRD), and X-ray fluorescence (XRF) analysis are used to justify the results. Experimental results show that incorporation of carbon dust in cement mortar production not only reduces its environmental side effects but also enhances the strength of cementitious composites. Up to 10% carbon dust by weight of cement can be added to the mixture without adversely affecting the strength of the mortar. Any further addition of carbon dust would decrease the strength. Best enhancement in compressive strength (27%) is achieved in the case of using 5% replacement ratio. SEM images show that incorporation of small amount of carbon dust (less than 10%) lead to produce denser and more compact-structure cement mortar.

## 1. Introduction

Growing industrialization and urbanization are recently noticed all over the world. This growing is associated with high production of industrial by-products. The huge amount of industrial by-products generated from the wide range of industries cause serious concerns to the environment and health. One of the industrial wastes is the carbon dust generated during the production of aluminum in the aluminum companies. Carbon dust is a by-product of anode manufacturing process usually generated during crushing of anode butts and cleaning of bath material during shot blasting process. It is super fine black powder. Aluminum companies usually generate large quantities of carbon dust. The carbon dust represents a main challenge to get rid of because of its environmental side effects such as air pollution due to its fineness, and the possible leaching to the groundwater. In addition, the carbon dust is usually dumped in landfills. The handling and transportation of the dust are also problematic. The high generation rate, the purity, and the finer particle size of this by-product lead to potential utilization in concrete production. 

Since the civil infrastructures around the world are mostly made of reinforced concrete (RC), the production and use of concrete increase rapidly. The high production and consumption rate of concrete make it a good option to recycle the industrial wastes. On the other hand, cement industry contributes about five to eight percent of the annual greenhouse gas emissions. The production of one ton of Portland cement generates about one ton of CO_2_ [1]. Cement replacement with supplementary cementitious materials (SCM) or industrial byproducts make it possible considerably to reduce the greenhouse gas emissions, reduce the environmental impacts, and decrease the consumption of natural resources. 

Various industrial by-products and solid wastes such as fly ash, slag, ceramic waste, bottom ash, granite dust, and marble dust are efficiently used in concrete production. The researchers studied this topic from different aspects. Some studies focused on the effect of using industrial byproducts on the strength of concrete. El-Dieb et al. [1] investigated the feasibility of using ceramic waste powder (CWP) to replace cement on concrete production. Their results indicated that CWP could be used to replace cement in concrete mixes and to enhance its behavior based on the replacement level. They concluded that partially replacement of cement by 10% CWP was suitable for strength enhancement, between 10% and 20% was adequate to enhance the workability while a 40% replacement was sufficient to improve the durability. Elahi et al. [2] investigated the mechanical and durability properties of high performance concretes containing SCM. Their results showed that silica fume performs better than other SCM used in the study for the strength development and bulk resistivity. Ali et al. [3] investigated the feasibility of using waste carbon black as a filler in producing lightweight concrete. They concluded that the lightweight concrete produced by substituting sand by carbon black could be used in both structural and non-structural purposes. Chitra et al. [4] showed that using carbon powder to replace cement enhanced the mechanical strengths of concrete and reduced its permeability. Schulze et al. [5] examined the ability of using natural calcined clay with different levels of quality as a cement constituent. Their results showed that natural calcined clays are suitable to be used as SCM in cement production.

Other studies focused on the effect of these materials on the durability of concrete. Ashish [6] studied the feasibility of using marble powder (MP) combined with SCM in concrete production. Two types of SCM namely silica fume and metakaolin were used to replace cement while the MP was used to replace sand. His results showed an enhancement in the durability of concrete because of 15% replacement of sand with MP combined with the use of SCM. Shah et al. [7] investigated the carbonation resistance of cements containing SCMs. Their results showed that the carbonation rate was ruled by the clinker replacement level, relative humidity and w/c ratio. Slag showed superior carbonation resistance ability compared to the other used SCMs. Farnam et al. [8] investigated the effect of SCM on damage caused by calcium oxychloride formation. They used several types of SCM to partially replace cement in cement paste production. Their results indicated that SCM improved the damage behavior of cementitious materials when exposed to CaCl_2_. Mangi et al. [9] studied the behavior of concrete with coal bottom ash (CBA) as SCM exposed to seawater. Their results showed that the compressive strength of concrete with SCM increased about 12% and 9% compared to control mix in water and seawater respectively at 180 days.

Other studies focused on the environmental aspect of waste utilization in concrete production. Zhang et al. [10] evaluated the environmental impact of concrete with SCMs using proposed integrated functional unit combining durability and compressive strength. The results revealed that adding fly ash or silica fume enhanced the environmental behavior compared to the ordinary concrete. Yang et al. [11] studied the feasibility of using various SCMs such as fly ash (FA), ground granulated blast-furnace slag (GGBS), and silica fume (SF) in reducing CO_2_ emissions from concrete. Their results showed that the intensity of CO_2_ decreased with increasing the dosage of the SCMs up to 15–20% replacement ratio. Vargas et al. [12] investigated the environmental impacts of using copper-treated tailings (CTT) as SCM. Their results showed that at higher mechanical behavior, CTT mixtures owned better environmental indicators than mixtures without CTT. Viet et al. [13] showed the ability of using fly ash (FA) generated from the thermal treatment solid waste as a CO_2_ sequester and as SCM to develop green construction materials. 

Other researchers focused on studying the feasibility of waste utilization in high strength concrete production. Pyo et al. [14] investigated the feasibility of using two types of quartz-based mine tailings to substitute silica powder and silica sand in ultra-high performance concrete (UHPC) production. They found that the shape and size of tailings particles affected the characteristics of the UHPC. They concluded that even though adding the tailings materials negatively affected the strength of the UHPC, these materials showed the capability to minimize the limitations due to the high production cost of the raw materials. Kim et al. [15] investigated the effect of using untreated coal bottom ash on the hydration kinetics of high-strength concrete. They found that incorporation of bottom ash in high-performance concrete for internal curing increased the degree of hydration in the cement matrix. 

The above-mentioned studies reflect that utilization of industrial wastes in concrete production, which is a massive construction material, is considered a good solution for solving the environmental impact of these materials. The high generation rate, the purity, and the finer particle size of carbon dust encourage the authors to investigate the feasibility of using it in cementitious composites production to partially replace cement. The aim of this study is to characterize the carbon powder generated as a by-product by aluminum factories with respect to its chemical composition, morphology, and particle size distribution. In addition, comprehensive study was conducted to evaluate the use of carbon powder as cement replacement on the strength and microstructure of cement mortar. 

## 2. Materials and Methods

### 2.1. Materials

Portland cement, fine aggregate, and tap water were used in this study to prepare the control cement mortar specimens. The cement was commercially available with chemical composition shown in Table 1. The fine aggregate was washed sand from a local supplier with properties listed in Table 2. The carbon dust was provided by Qatar Aluminum Company (Qatalum, Doha, Qatar). It is super fine black talcum powder with a density of 1.9 g/cm^3^. The high generation rate, the purity, and the finer particle size of this waste by-product lead to potential utilization in concrete production. It was used as-received from Qatalum. Comprehensive characterization was performed to explore its properties as shown in Section 3.1.

### 2.2. Carbon Dust Characteristics

The main properties of carbon dust used in this study such as particle size distribution, particles’ shape, morphology, and chemical composition were investigated. The chemical composition analysis was conducted through X-ray diffraction (XRD) and X-ray fluorescence (XRF) test procedures. The carbon dust was sieved to pass through a 325-mesh sieve. The powder was placed on the JSX 3201M (Jeol) spectroscopy machine (JEOL, Peabody, MA, USA) to conduct the elemental tests. The morphology of the carbon dust was examined by scanning electron microscopy (SEM) technique using NOVA NanoSEM 450 device (Hillsboro, OR, USA) at high voltage of 5 kV and 20 kV and working distance ranging from 4.6 to 9 mm.

### 2.3. Mix Design and Test Specimens 

Mortar mixes with different amounts of carbon dust were cast. Five replacement levels by weight of cement were studied 5%, 10%, 20%, 30%, and 40%. The mixtures were identified with the letters CD and two number, the letters refer to the carbon dust, and the numbers refer to the replacement ratio and the age of the specimen at the time of testing, respectively. For example, CD10-28 represents the mixture with 10% replacement ratio of carbon dust tested at 28 days. Table 3 summarizes the mix proportions and specimens designations. ASTM standard C305 was followed to mix and cast the cement mortars. For compressive strength test, the cement mortar was mixed and cast in 50-mm cube molds. Twenty-four hours after casting, the specimens were demolded and cured in lime-saturated water for different periods until the time of testing. 

### 2.4. Test Methods

**Figure 1:**
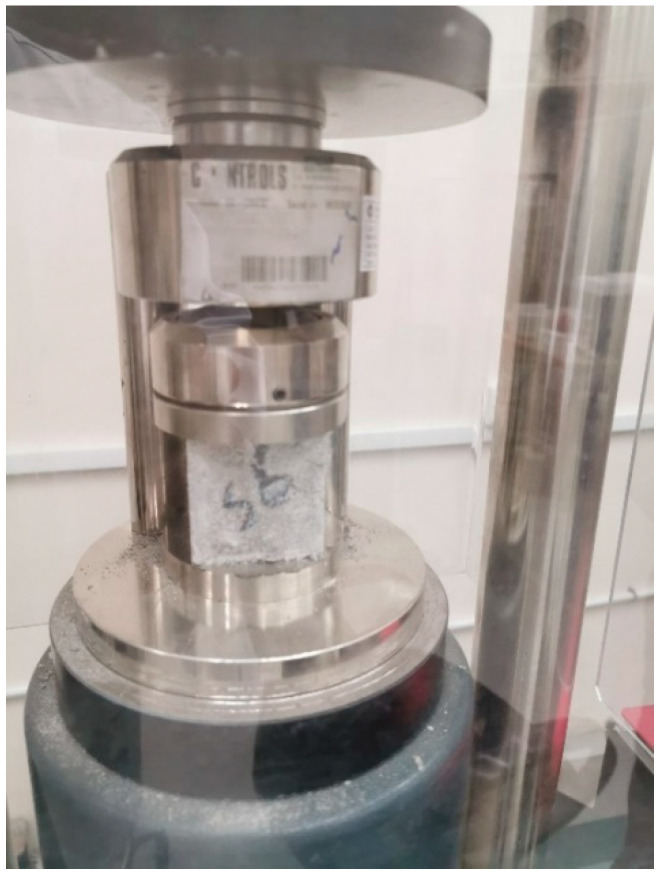
Compressive strength test setup.

The effect of carbon dust on the development of compressive strength of cement mortar with age was studied. Compressive strength test was performed after 3, 7, and 28 days of curing. Three specimens were tested for each mix at each age according to the ASTM 109/C109M. The test was performed using universal testing machine with loading rates of 1.3 kN/s. The compressive strength test setup is shown in Figure 1. 

At the end of the compressive strength test, scanning electron microscopy (SEM) imaging test was performed on selected specimens to explore the effect of carbon dust on the microstructure and morphology of cement mortar. Small fragments were extracted from different locations of the selected specimens to represent the entire sample. To enhance the conductivity of the mortar fragments, the surface of the fragments was coated with gold. After coating, the SEM analysis was conducted according to the ASTM C1723-10 using NOVA NanoSEM 450 device at high voltage of 5 kV and 20 kV and working distance ranging from 4.6 to 9 mm. 

In addition, X-ray fluorescence (XRF) and X-ray diffraction (XRD) tests were conducted to investigate the effect of carbon dust on the chemical composition of cement mortar. Selected cement mortar specimens were crushed into size that can pass sieve size 150 µm. To make sure that the results are representative, the powder of each specimen was carefully mixed, and enough amount was taken to run the test. The powder was then placed on the JSX 3201M (Jeol) spectroscopy machine to conduct the analysis. 

## 3. Results and Discussion

### 3.1. Carbon Dust Characteristics 

**Figure 2:**
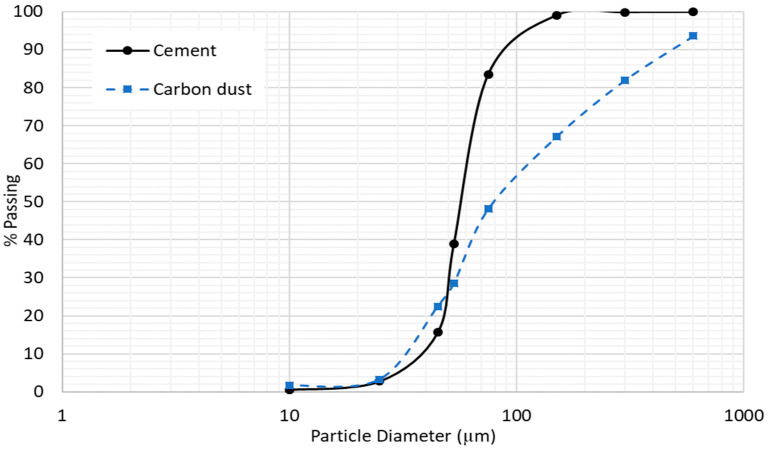
Particle size distribution of cement and carbon dust.

**Figure 3:**
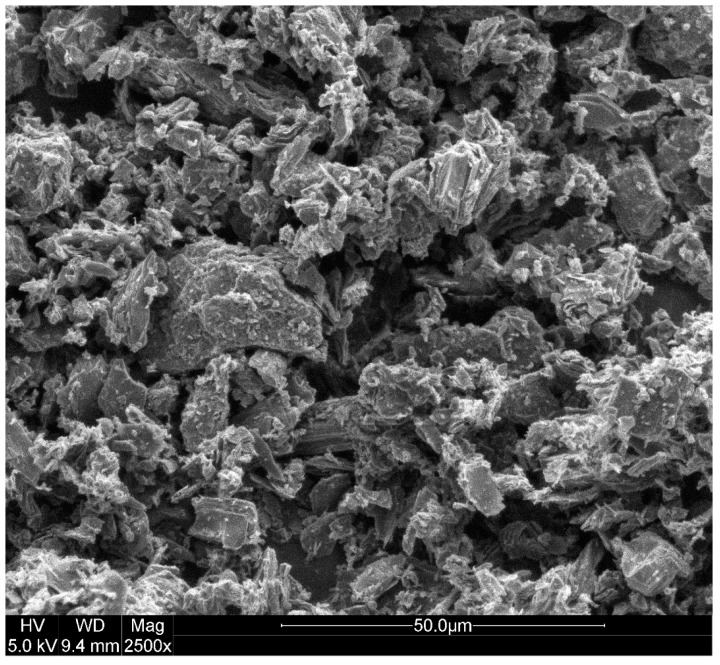
SEM image of carbon dust particles.

The particle size distribution (PSD) of cement and carbon dust are shown in Figure 2. The figure reveals that both cement and carbon dust have continuous graded PSDs. Some of the carbon dust particles are finer than the cement particles, whereas some other particles are coarser. Almost 20% of the carbon dust particles are smaller than 30 μm, whereas 50% of the particles are smaller than 80 μm. SEM images of the carbon dust show that it has angular and irregular shapes and very small size particles (powder), as shown in Figure 3. 

**Figure 4:**
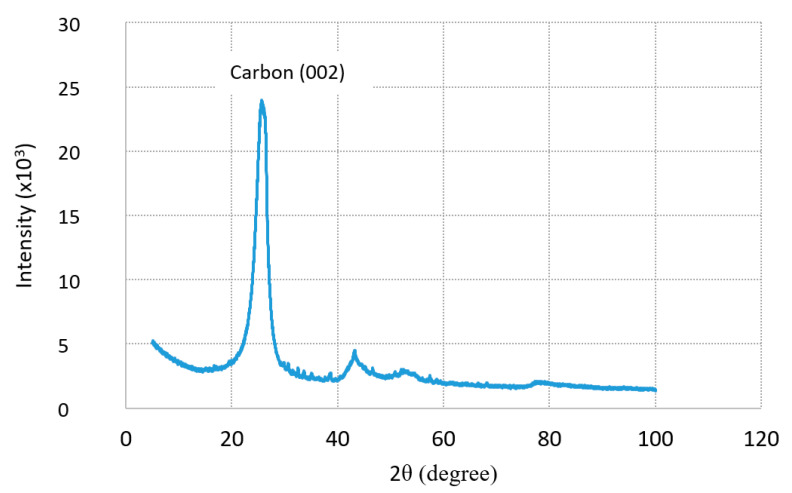
XRD pattern of carbon dust.

Table 4 summarizes the results of the chemical composition analysis of the carbon dust using XRF analysis. It is clear that the carbon dust is mainly composed of carbon. It presents almost 85% of the total mass. Moreover, very small percentages of other materials such as iron, fluoride, and sodium are observed. The mineralogical composition of carbon dust was investigated using XRD analysis. It is clear from the XRD patterns shown in Figure 4 that the carbon dust shows a very broad carbon peak at 2θ = 25° that can be attributed to two different forms of carbon, turbostratic carbon (carbon black) and graphene carbon (graphitic structure) [16,17]. These results support the XRF results mentioned previously. 

### 3.2. Compressive Strength Results

**Figure 5:**
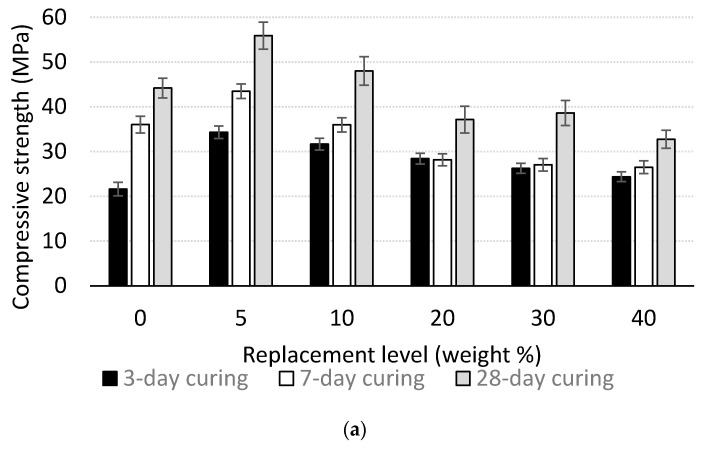

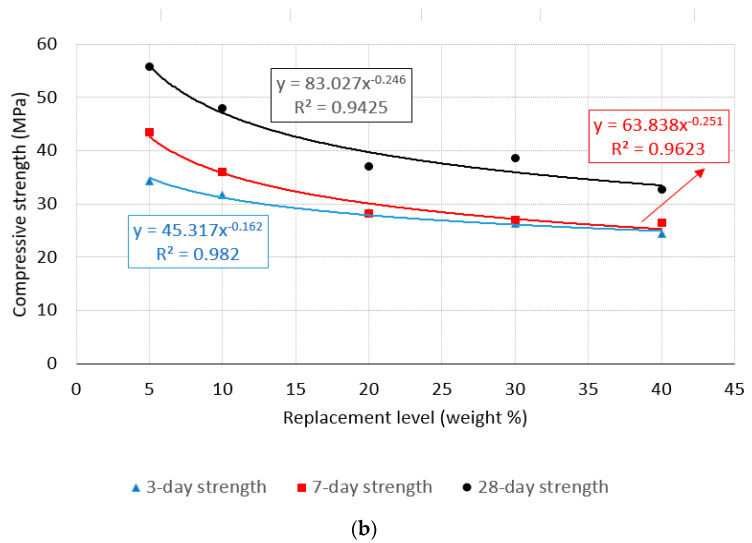
Compressive strength development for mortar with different replacement ratios; (**a**) experimental results, (**b**) best fit curves.

The influence of carbon dust on the compressive strength of cement mortar at different ages is shown in Table 5 and Figure 5. It is clear that incorporation of carbon dust significantly enhances the compressive strength of cement mortar at early age (3-day strength) for all replacement ratios 5%, 10%, 20%, 30%, and 40% compared to the control specimen. The highest enhancement in the 3-day strength is achieved in the case of 5% replacement ratio with about 59% compared to the control specimen. The enhancement in the compressive strength of the mortar at early age because of carbon dust incorporation could be attributed to the micro-filling ability of the carbon dust because of its small particle size [1,18]. For the 28-day strength, partially replacing cement with 5% and 10% carbon dust enhances the compressive strength of the mortar by 27% and 9%, respectively. For replacement ratio of 20% and higher, incorporation of the carbon dust reduces the compressive strength of the mortar. Similar results are reported in other studies [1,4,6]. The enhancement in the 28-day compressive strength of the mortar due to carbon dust incorporation could be attributed to many reasons: (1) The micro-filling ability (filler effect) of the carbon dust that helped in forming a denser mixture [1,6,19,20] and improved the transition zone and cement matrix property [21]; (2) the carbon dust particles worked as nucleation spots for the hydration products [1]. The reduction in compressive strength when using high replacement ratio of carbon dust could be attributed to the following reasons: (1) The dilution effect where replacing cement with carbon dust negatively affect the strength development; (2) the micro-filling effect of carbon dust could not balance the reduction in the cement content [1]. 

### 3.3. Correlation between Compressive Strength and Replacement Ratio

Statistical analysis is very helpful in analyzing the variation of the experimental results. For this purpose, the standard deviation and regression analysis are used to investigate the trending of the results. The standard deviation for compressive strength of specimens contain various dosages of carbon dust and cured for different periods are listed in Table 5. According to the standard deviation values, the variability of the data is considerably small. The correlations between the carbon dust dosages and the compressive strength for specimens cured at various periods are shown in Figure 5b. Best-fit curves determined according to the data of each curing period are also presented in the figure. The results reveal that satisfactory power relationship exist between the dosage of the carbon dust and the compressive strength values for all curing periods. In addition, the R-square values of 0.98, 0.96, and 0.94 reflect the strong correlations between the compressive strength and the carbon dust replacement ratio for specimens cured for 3, 7, and 28 days, respectively. 

### 3.4. Chemical Composition of Cement Mortar

The effect of carbon dust replacement ratio on the chemical composition of cement mortar at 28-day age was investigated through XRF analysis. The results are summarized in Table 6. It is clear that for 5% replacement ratio, the CaO percentage decreased and the SiO_2_ percentage increased, compared to the control specimen. This finding reflected the reduction in CH and replacement of C-S-H in the case of carbon dust addition. As the replacement ratio increased to be 10%, the CaO percentage increased compared to the 5% specimen but still below the value of the control specimen. In addition, the SiO_2_ percentage decreased compared to the 5% specimen but still above the value of the control specimen. For higher replacement ratios such as 20%, the CaO percentage increased above the control specimen and the SiO_2_ percentage decreased below the control specimen. These results could be used to clarify the compressive strength test results reported in the previous section. 

The CaO/SiO_2_ ratio is usually used to compare the quality of hydration. It is clear in Table 6 that the CaO/SiO_2_ ratio for specimens with 5% and 10% replacement ratios is less than that of the control specimen. The reduction in the ratio reflected more consumption of the CH and more formulation of the C-S-H in the presence of carbon dust. Furthermore, the lower ratio of CaO/SiO_2_ indicated the suitable quantities of C3S and C2S in the microstructure, thus improved strength development [22]. This result explains the enhancement in the compressive strength of the mortar with 5% and 10% replacement ratio compared to the control specimen.

### 3.5. Microstructure of Cement Mortar 

**Figure 6:**
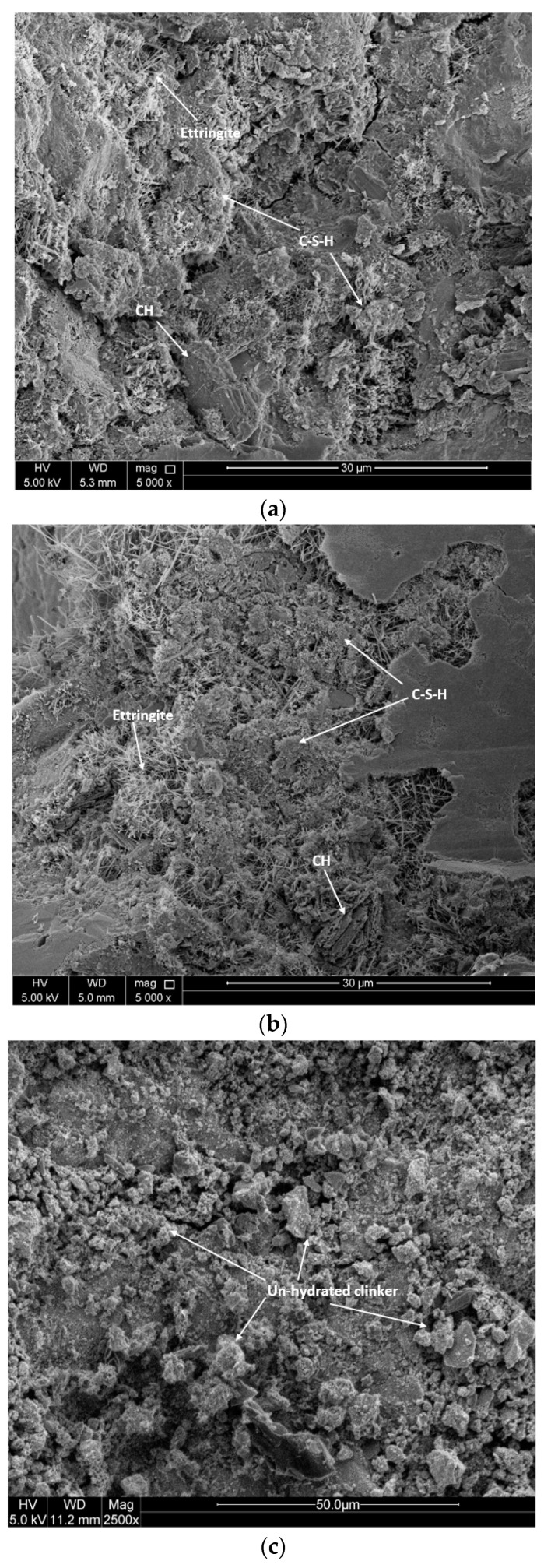
SEM images for selected mortar specimens with (**a**) 0% carbon dust, (**b**) 5% carbon dust, (**c**) 40% carbon dust.

To investigate the effect of carbon dust on the microstructure of cement mortar, scanning electron microscopy (SEM) imaging was conducted. Figure 6 shows the microstructure of mortar specimens with different amount of carbon dust cured for 28 days. The SEM micrograph of control specimen made without carbon dust reflects the formation of the main hydration products such as calcium hydroxide (CH) platelets, calcium silicate hydrate (CSH), and calcium sulphoaluminate hydrate (Ettringite) needles as shown in Figure 6a. The SEM micrograph of specimens with low replacement ratio such as 5% and 10% carbon dust reflects the formation of more dense and compact structure with the presence of well-formed and dense clusters of C-S-H as compared to that of control specimen as shown in Figure 6b. The dense structure could be mainly attributed to many reasons such as (1) the formation of an excessive amount of CSH with consumption of CH in the present of carbon dust. These hydration products filled the microspores and resulted in less porous and denser structure [1]. (2) The carbon dust particles served as nucleus for hydration products [4,23]. This observation supports the compressive strength results. In addition, the ettringite needles are revealed to be in abundance. The well-formed ettringite crystals signal toward the healthy mix of cement mortar [22]. 

Figure 6c shows the microstructure of cement mortar specimen with high replacement ratio of carbon dust (40%). The microstructure of this specimen looks different than the previous ones, which explains the reduction in its compressive strength. It is clear that the clinker-shaped particles of CH that appears like the structure of un-hydrated clinkers of cement. The clusters of un-hydrated clinkers of Portlandite is formed due to the unavailability of water for proper hydration of concrete. Adding large amount of carbon dust led to absorb some of the water that should be used to complete the hydration process. In addition, the micro-filling effect of carbon dust could not balance the reduction in the cement content. The un-hydrated clinker particles have lower bonding capacity than well hydrated C-S-H and ettringite, thus, reducing the strength of the mortar.

### 3.6. Cement Content and Mortar Sustainability 

**Figure 7:**
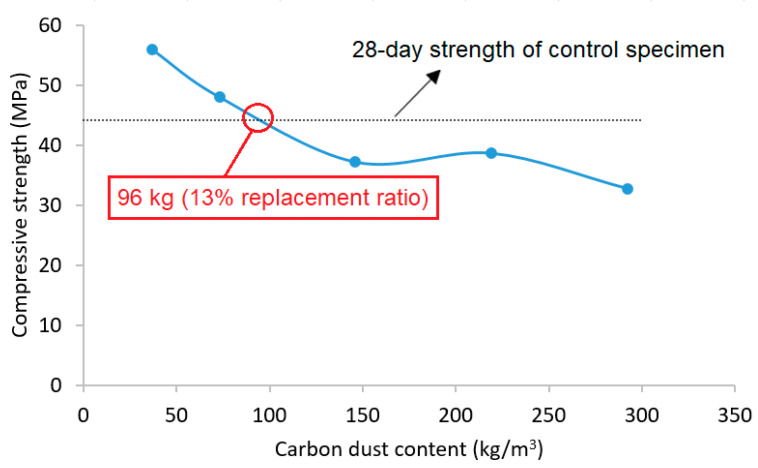
Carbon dust content versus compressive strength of mortar.

To connect between the simultaneous changes in cement content and strength of cement mortar, Figure 7 shows the variation in the 28-day compressive strength of cement mortar with various dosages of carbon dust. The horizontal line represents the 28-day strength of control specimen (without carbon dust). The figure reveals that maximum of 96 kg/m^3^ of cement content could be replaced with carbon dust (equivalent to 13% replacement ratio) without negatively affecting the 28-day compressive strength of the mortar. Any further cement replacement by carbon dust would decrease the strength to be less than that of control specimen. So, the filler technology of adding carbon dust to decrease the cement content in mortar mix could be applicable to certain limit without negatively affecting its strength.

The recycling of carbon dust in cement mortar production has four major benefits in order to produce sustainable construction materials. First, replacing cement by carbon dust reduces the amount of cement needed for mortar production. That will significantly reduce the carbon footprint of cement mortar production since the major contribution of carbon footprint (about 90%) comes from cement production. Second, replacing cement with carbon dust reduces the cost of concrete due to the high price of cement compared with carbon dust (which is waste materials). Third, using carbon dust in cement mortar production thus in concrete would considerably reduce the amount of carbon dust that is disposed to landfills because of the huge consumption of concrete as construction material. That will extend the life period of limited capacity landfill used to store these wastes thus mitigate the environmental issues with difficulties to find new sites for landfills. Finally, replacing cement by carbon dust will not only improve the environmental sustainability but also enhance the compressive strength of cement mortar. However, it is important to run comprehensive durability study to investigate the effect of carbon dust on the durability properties of cement mortar and concrete. 

## 4. Conclusions

The effect of utilization of carbon dust generated as an industrial waste from aluminum factories in cement mortar production was studied in this research. Experimental program was conducted to characterize the carbon dust, and then to investigate the effect of partially replacement of cement by carbon dust in the strength, microstructure, and chemical composition of cement mortar. The following conclusions could be drawn:

1. Partially replacing cement with small amount of carbon dust (5% and 10%) enhanced the compressive strength of the mortar. The maximum enhancement was 27% for 5% replacement ratio. For higher replacement ratio (more than 20%), incorporation of the carbon dust reduced the compressive strength of the mortar.
2. For low replacement ratio (5% and 10%), incorporation of carbon dust led to the formation of more dense and compact structure with the presence of well-formed and dense clusters of C-S-H as compared to the control specimen.
3. The microstructure of cement mortar with high replacement ratio (more than 20%) of carbon dust showed large quantities of un-hydrated clinkers reflecting improper hydration of concrete.
4. The maximum reduction in cement content that can be considered without negatively affecting the 28-day strength of the mortar was 96 kg/m^3^ (equivalent to 13% replacement ratio). Further cement replacement would decrease the strength.
5. Adding carbon dust as cement replacement would help in reducing the carbon footprint associated with concrete production and help in extending the life period of limited capacity landfill used to store these wastes thus mitigating the related environmental issues.

## Figures and Tables

**Table 1:** Constituents of cement.

Compound Name	Content Percentage
**CaO**	6.4%
**SiO_2_**	18.4%
**Fe_2_O_3_**	6.1%
**SO_3_**	3.0%
**Al_2_O_3_**	2.2%
**MgO**	1.4%
**Na_2_O**	0.8%
**LOI**	1.7%

**Table 2:** Fine aggregate properties.

Material	Fineness Modulus	Specific Gravity	Density (kg/m^3^)	Water Absorption (%)	Moisture Content (%)
Sand	2.31	2.564	2558.3	1.87	3.00

**Table 3:** Mix proportions and specimen’s designation.

Specimen Designation	CD0	CD5	CD10	CD20	CD30	CD40
Replacement ratio (%)	0	5	10	20	30	40
Cement (kg/m^3^)	731	694	658	585	512	439
Carbon powder (kg/m^3^)	0.0	37	73	146	219	292
Sand (kg/m^3^)	2010	2010	2010	2010	2010	2010
Water (kg/m^3^)	355	355	355	355	355	355

**Table 4:** XRF analysis of carbon dust.

Name	Concentration (%)
Carbon	85
Iron	7
Fluoride	4
Sodium	2.5
Sulphur	1
Silicon	0.5

**Table 5:** Compressive strength results.

Specimen		CD0	CD5	CD10	CD20	CD30	CD40
Replacement %		0%	5%	10%	20%	30%	40%
3-day curing	Strength (MPa)	21.6	34.3	31.7	28.4	26.3	24.4
	Standard deviation (±)	1.5	1.7	1.4	1.3	1.2	1.1
	Enhancement (%)	NA	58.9	46.6	31.6	21.5	12.9
7-day curing	Strength (MPa)	36.0	43.5	36.0	28.2	27.0	26.5
	Standard deviation (±)	1.9	2.0	1.6	1.6	1.35	1.4
	Enhancement (%)	NA	20.8	−0.1	−21.8	−24.9	−26.4
28-day curing	Strength (MPa)	44.2	55.9	48.0	37.2	38.6	32.7
	Standard deviation (±)	2.2	2.0	3.0	3.2	3.0	2.8
	Enhancement (%)	NA	26.5	8.6	−16.0	−12.6	−25.9

**Table 6:** XRF analysis of mortar with carbon dust.

Replacement Ratio	0%	5%	10%	20%
SiO_2_	58.5	62.6	60.1	52.3
CaO	31.5	26.3	29.8	34.5
Fe_2_O_3_	3.3	4.5	5.9	3.6
SO_3_	1.9	1.8	3	2.6
Al_2_O_3_	2.3	2.5	2.2	3.4
K_2_O	0.6	0.8	0.4	0.6
Na_2_O	1.1	0.7	1.8	1.8
Ca/SiO_2_	0.54	0.42	0.50	0.66
